# Central Line-Related Bloodstream Infection by *Saccharomyces cerevisiae* Following Probiotic Use in a Patient with *Clostridioides difficile* Colitis: A Case Report

**DOI:** 10.3390/microorganisms14010070

**Published:** 2025-12-29

**Authors:** Yu-Mi Lee

**Affiliations:** Department of Infectious Diseases, Kyung Hee University Hospital, Kyung Hee University College of Medicine, 23, Kyungheedae-ro, Dongdaemun-gu, Seoul 02447, Republic of Korea; cristal156@hanmail.net; Tel.: +82-2-958-8209; Fax: +82-2-968-1848

**Keywords:** central venous catheter, *Clostridioides difficile*, fungemia, probiotics, *Saccharomyces cerevisiae*

## Abstract

Clinical infections caused by *Saccharomyces cerevisiae* develop infrequently. We report a case of central line-related bloodstream infection caused by *S. cerevisiae* in a patient with *Clostridioides difficile* colitis and central venous catheter placement. The administration of probiotics in *C. difficile* colitis was associated with fungemia caused by *S. cerevisiae*. This case suggests the potential for serious infection caused by *S. cerevisiae* in immunocompromised patients with *C. difficile* colitis. Removal of the central venous catheter may be important for a better prognosis in fungemia caused by *S. cerevisiae*.

## 1. Introduction

*Saccharomyces cerevisiae*, also called brewer’s yeast, is ubiquitous in plants and in soil [1]. It plays a critical role in numerous food fermentation processes and industrial applications. *Saccharomyces boulardii* is a subtype of *S. cerevisiae*, which is utilized as a probiotic [2]. Probiotics are consumed by many healthy individuals to maintain gut health and are also used for therapeutic purposes in cases of diarrhea or intestinal diseases [3]. Many studies have shown that the use of probiotics can be beneficial for treating and preventing the recurrence of diarrhea in patients with *Clostridioides difficile*, leading to the frequent administration of probiotics with antibiotics [4,5,6].

Probiotics, which are used for their various benefits, can pose risks such as fungemia in certain cases. There have been some reports of bloodstream infection caused by *S. cerevisiae* [1,7]. However, central line-associated bloodstream infections (CLABSIs) caused by *S. cerevisiae* have not been emphasized, particularly in patients with *C. difficile* colitis. We present a case of catheter-related bloodstream infection (CRBSI) caused by *S. cerevisiae* following probiotic use in a patient with *C. difficile* colitis. This study was approved by the Institutional Review Board (KHUH-2025-06-033) of Kyung Hee University Hospital, Seoul, Korea, which waived the need for written informed consent from the patient.

## 2. Case Presentation

A 74-year-old male patient presenting with voiding difficulty was admitted to the Pulmonology Department of Kyung Hee University Hospital in South Korea in August 2024. The patient had chronic obstructive pulmonary disease and benign prostatic hyperplasia. The patient was a hepatitis C carrier. Two years prior, the patient was diagnosed with non-small cell lung cancer (NSCLC) [cT4N1M0, stage IIIA]. The patient underwent neoadjuvant chemotherapy with three cycles of nivolumab, carboplatin, and paclitaxel, followed by left lower lobectomy. Seven months after surgery, NSCLC recurred with bone and pleural metastasis (cT4N0M1c). The patient had received chemotherapy with pembrolizumab, paclitaxel, and carboplatin one month prior to admission, and also underwent palliative radiation therapy at the C7 cervical spine level ten times starting one month before admission.

Ten months prior to admission, the patient was diagnosed with drug-induced immune-mediated colitis, probably caused by nivolumab. Since then, probiotics containing *S. boulardii* have been administered to the patient for 3 months and then readministered for 25 days prior to admission, continuing up until the time of admission. The patient underwent chemoport insertion in the right anterior chest wall for chemotherapy 37 days prior to admission. The initial vital signs were blood pressure, 107/66 mmHg; pulse rate, 100 beats/min; respiratory rate, 18 breaths/min; temperature, 36.4 °C, pulse oximetry, 98% on room air. Laboratory examination revealed a white blood cell count of 3.51 × 10^9^/L (58% neutrophils) and C-reactive protein (CRP) was 7.77 mg/dL. The results of the kidney and liver function tests were within normal limits. Chest computed tomography showed no interval change in the left pleural thickening or a metastatic mass in the left upper lobe. The probiotics were continuously administered following admission.

On the 2nd day of hospitalization, the patient developed watery diarrhea, occurring approximately ten times a day, with a total volume of approximately 200 g. The patient had no fever. Laboratory examination revealed a white blood cell (WBC) count of 29.83 × 10^9^/L (88% neutrophils) and CRP was 27.1 mg/dL. The *C. difficile* toxin-polymerase chain reaction (PCR) was positive for Toxin B with a cycle threshold value of 24.6. Toxin A + B of *C. difficile* were also positive. For molecular detection, we used Xpert *C. difficile* (Cepheid, Sunnyvale, CA, USA), which targets the *tcdB* gene and binary toxin genes. The *C. difficile* toxin antigen detection was performed using VIDAS *C. difficile* Toxin A & B (bioMérieux, Marcy l’Etoile, France). Three sets of blood cultures were performed on the 2nd day of hospitalization. The patient was initially prescribed meropenem (3 g per day) and oral vancomycin (500 mg per day). On the 6th day of hospitalization, one set of aerobic cultures identified *Acinetobacter nosocomialis*. Watery diarrhea improved, and the inflammatory marker, CRP, also decreased to 1.74 mg/dL. On the 12th day of hospitalization, three sets of blood cultures were performed using BD Bactec Plus Aerobic/F and BD Bactec Plus Anaerobic/F bottles and aBactec FX Instrument (Becton Dickinson, Sparks, MD, USA) because the CRP level had increased again to 4.94 mg/dL.

**Figure 1:**
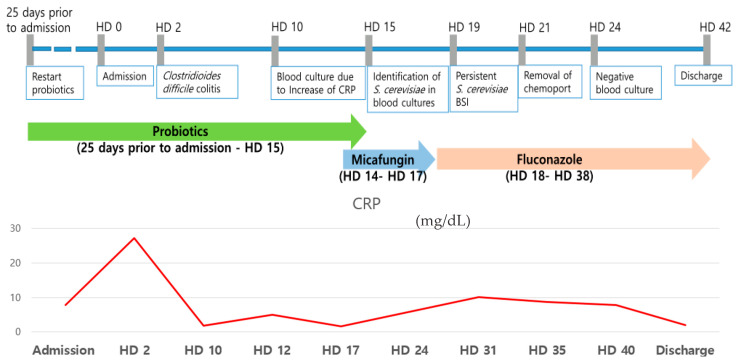
Clinical course and antifungal treatment of a case patient. HD, hospital day, CRP, C-reactive protein, BSI, bloodstream infection.

The three sets of aerobic culture bottles identified *S. cerevisiae* on the 15th day of hospitalization. *S. cerevisiae* was identified using a MALDI-TOF MS system (Bruker Daltonics, Bremen, Germany) following colony pretreatment with the extended direct transfer method. A small amount of a fresh colony was smeared onto a target plate and overlaid with 1 µL of 70% formic acid. After drying, 1 µL of matrix solution (α-cyano-4-hydroxycinnamic acid, HCCA) was added and allowed to air-dry before analysis. The analysis yielded a score of 2.0, as measured by the IVD MALDI Biotyper software version 4.2 with IVD Library version 10.0. 18S rRNA sequencing analysis revealed that the fungal sequence had a 100% match with *S. cerevisiae* (GenBank accession number MG101837.1), with the alignment showing identical (1652/1652) base pairs. The patient was initially administered micafungin (100 mg per day) for 4 days. The same organism was grown in subsequent blood cultures from three sets of bottles performed on the 15th and 19th day of hospitalization. The chemoport was removed on the 21st day of hospitalization, and *S. cerevisiae* was isolated from the chemoport tip culture (>15 colony-forming units). The antibiotic therapy was changed to fluconazole (400 mg per day) for 22 days. The probiotics were discontinued after *S. cerevisiae* was identified. No organisms grew in the following blood cultures with three sets of bottles performed on the 24th day of hospitalization. No fever was observed during the treatment. At the end of treatment, the WBC count returned to within the reference interval, and the CRP level was 1.9 mg/dL. The patient did not experience a recurrence of *C. difficile* colitis during the 6-month follow-up period after discharge. Antifungal susceptibility testing was performed after the completion of treatment. The minimum inhibitory concentration (MIC) of antifungal drugs was tested using the Vitek 2 AST-YS08 card, which is designed for yeast susceptibility testing. The MICs of fluconazole, caspofungin, and flucytosine were 32 µg/mL, 0.25 µg/mL, and ≤1 µg/mL, respectively. The susceptibility of voriconazole and amphoterin B were not assessed due to insufficient growth in the positive control well. Antifungal susceptibility testing for *S. cerevisiae* was not routinely performed in our hospital. The patient was administered fluconazole despite the high MIC because the antifungal susceptibility testing was conducted after the completion of treatment. The clinical course and antifungal treatment of the case patient are shown in Figure 1.

## 3. Discussion

*S. cerevisiae* is a facultative anaerobic fungus that is genetically simple and contains approximately 6000 genes [1,8]. It primarily undergoes fermentation under anaerobic conditions to produce ethanol and carbon dioxide, which are crucial for brewing and baking [9]. This species is essential for both industrial applications and scientific research because of its ease of cultivation and straightforward genetic modifications [10]. The identification of *S. cerevisiae* is not particularly difficult in laboratories that use microbiological and molecular equipment. *S. cerevisiae* can be identified by observing round-to-oval budding yeast cells under a microscope [11]. It forms creamy and smooth colonies on culture media. *S. cerevisiae* typically grows well at 30 °C and cannot grow at higher temperatures [11,12]. Molecular methods such as polymerase chain reaction targeting specific genetic markers and DNA sequencing, and MALDI-TOF MS can also be used to accurately identify *S. cerevisiae* [13,14]. *S. cerevisiae* and *S. boulardii* are taxonomically similar. It is challenging to differentiate them using typical laboratory tests. Molecular analysis is needed to distinguish them [15].

*S. cerevisiae* has a low potential to cause clinical infections. However, *S. cerevisiae* can cause invasive infections under certain conditions, particularly in immunocompromised or critically ill patients, such as those with malignancy receiving chemotherapy, human immunodeficiency virus infection, chronic renal failure, antibiotic use, total parenteral nutrition, and undergoing organ transplants [1,7,16,17,18,19]. Admission to the intensive care unit with critical illness and prolonged hospitalization are risk factors for *S. cerevisiae* bloodstream infection [7]. Munoz et al. reported that of 44 adult patients, all but 3 had underlying conditions, with 25 (57%) being immunocompromised. In this study, 60% were intensive care unit (ICU) patients [1]. Eric et al. revealed that 14 (78%) out of 18 patients were in the ICU [7]. The unique aspect of our report is that it focuses on the occurrence of bloodstream infections due to *S. cerevisiae* in patients with *C. difficile* colitis who frequently take probiotics, and highlights that CRBSIs can occur due to this organism in patients with central venous catheters.

Probiotics are typically prescribed to patients with *C. difficile* colitis for several benefits [5]. Probiotics aid in the early treatment of *C. difficile* colitis by enhancing intestinal immunity [20]. They also help to prevent the recurrence of *C. difficile* colitis. *S. boulardii* in probiotics secretes a protease that degrades the intestinal receptor for *C. difficile* toxins and breaks down the toxin molecule [21]. This protease suppresses ileal secretion, mannitol permeability, and toxin-induced histological damage. However, fungemia caused by *S. cerevisiae* can develop when probiotics are administered to patients with *C. difficile* colitis. *C. difficile* colitis may be linked to microbial translocation and intestinal injury, which persist even after the clinical resolution of *C. difficile* colitis [22]. Hasegawa et al. reported the translocation of commensal bacteria across the intestinal epithelial barrier in *C. difficile* infection, which is suspected to be caused by the weakening of IL-1β medicated neutrophil recruitment [23]. This weakening induces the translocation of bacteria to organs after *C. difficile* infection. The translocation of *S. cerevisiae* can cause bloodstream infections due to a weakened intestinal barrier caused by *C. difficile* colitis, especially in immunocompromised hosts. There have been previous reports of fungemia caused by *S. cerevisiae* in patients who were administered probiotic preparations containing *S. boulardii* [1,24,25]. The incidence of bloodstream infections caused by *S. cerevisiae* associated with the administration of probiotics was low as 1.70 cases per 10,000 patient days and 0.26 cases per 1000 central-line days [7]. There have been a few reports of such cases following *C. difficile* colitis (Table 1). Santino et al. documented a case of fungemia caused by *S. cerevisiae* related to probiotic use in a patient with chronic obstructive pulmonary disease who developed *C. difficile* colitis [17]. In this case, the central line culture was negative. Munoz et al. reported that 53.3% of 60 cases of fungemia caused by *Sacharomyces* occurred after probiotic use at a median interval of 10 days [1]. In our case, the patient had been taking probiotics containing *S. boulardii* while suffering from *C. difficile* colitis. *S. cerevisiae* may have translocated across the impaired intestinal mucosal barrier into the bloodstream. Subsequently, the circulating *S. cerevisiae* likely adhered to and colonized the indwelling central venous catheter, leading to a CRBSI. This scenario was supported by the isolation of *S. cerevisiae* from both peripheral blood and the catheter tip, as well as the resolution of fungemia only after the catheter was removed.

Central lines are frequently used to administer medications and monitor in immunocompromised and critically ill patients. CRBSI caused by *S. cerevisiae* may occur. Fungemia caused by *S. cerevisiae* can develop regardless of probiotic use [26,27]. In such situations, the central line can serve as a major source of infection [16]. Previous studies have not specifically focused on CRBSI caused by *S. cerevisiae*. We investigated central line-associated BSI caused by *S. cerevisiae*, which can be seen in Table 2. There were four cases of CRBSI, and one patient died despite the removal of the central line. One case of fungemia caused by *S. cerevisiae*, a patient who did not have the central venous catheter removed did not survive despite receiving appropriate antifungal therapy [27]. Among 15 patients whose central venous catheters were removed, 5 died. Our patient experienced resolution of bloodstream infections caused by *S. cerevisiae* following the removal of the central venous catheter. This case highlights the importance of the early removal of central venous catheters in patients with BSIs caused by *S. cerevisiae* and central venous catheter placement. Biofilm formation is a key factor in the pathogenesis of CRBSI. *S. cerevisiae* forms biofilms similar to *Candida* species, making them difficult to eradicate and potentially leading to antifungal resistance. Li et al. reported that the presence of central venous catheters and stronger biofilm forming strains of *Candida* species were independent risk factors for persistent candidemia [28]. Boisen et al. reported that only amphotericin B was effective in reducing the viable cell number when treating mature biofilms [29]. They discovered that cells within mature biofilms exhibit slower metabolism and reduced growth, as visualized using FUN-1 staining. Cells with stagnant growth in mature biofilms were resistant to antifungal drugs with the exception of amphotericin B. This study suggested that if *S. cerevisiae* forms a biofilm on a central venous catheter, it may be difficult to treat with antifungal agents alone without removing the catheter.

*S. cerevisiae* can persist in hospital environments, leading to cross-contamination and outbreaks of infection. There are cases in which *S. cerevisiae* fungemia can occur in patients taking probiotics in close proximity within a hospital environment. Olver et al. evaluated the nosocomial transmission of *S. cerevisiae* infections among bone marrow transplant patients at the hematology unit [30]. Screening test for *S. cerevisiae* in throat and stool samples revealed colonization in four patients. Genetic testing confirmed that the strains were identical to those of patients who shared the same ward at the same time, indicating cross-infection. Although the exact transmission route was not analyzed in this case, it suggests that *S. cerevisiae* may be transmitted through contact of airborne routes in hospital settings. Cassone et al. reported an outbreak case of bloodstream infections caused by *S. cerevisiae* in the ICU, where the central venous catheter was probably contaminated during the probiotic preparation by nurses [26]. Therefore, it is important to recognize the possibility of *S. cerevisiae* fungemia outbreaks in patients taking probiotics or during their probiotic preparation, and to pay attention to infection control measures, including hand hygiene, to prevent the cross-infection of *S. cerevisiae*.

**Table 1:** Characteristics of patients with *Saccharomyces cerevisiae* fungemia in *Clostridioides difficile* colitis patients.

Patient	Age/ Sex	ICU Stay	Underlying Disease and Medical Condition	Probiotic Use	Central Line	Previous Antibiotic Use	Other Positive Culture Specimen	Time to Fungemia (Days) ^a^	Co-Pathogen	Central Line Removal	Treatment	Outcome	Reference
1	M/86	NR	Chronic obstructive bronchitis	Yes	Yes	NR	None	10	None	Yes	Caspofungin	Survived	[17]
2	F/72	NR	Undergone heart surgery	Yes	NR	Yes	None	7	None	NR	No therapy	Died	[1]
3	F/74	NR	Rheumatoid arthritis Undergone heart surgery	Yes	Yes	Yes	None	7	None	Yes	Fluconazole	Died	[1]
4	F/76	NR	Undergone heart surgery, myocardial infarction	Yes	NR	Yes	None	8	None	NR	Fluconazole	Died Endocarditis	[1]
5	F/51	NR	Polyarteritis nodosa	Yes	NR	Yes	None	18	None	NR	Amphotericin B	Survived	[31]
6	F/42	NR	Undergone kidney-pancreas transplantation	Yes	NR	Yes	None	7	None	NR	Fluconazole	Survived	[32]
7	M/41	NR	HIV, Tuberculous meningitis	Yes	NR	NR	None	15 days after discontinuation	None	NR	Amphotericin B	Survived	[32]

M, male; F, female; ICU, intensive care unit; NR, not reported; HIV, human immunodeficiency virus. ^a^ Time from the start of probiotic use to the development of *Saccharomyces cerevisiae* fungemia.

**Table 2:** Characteristics of patients with central line-associated with bloodstream infections caused by *Saccharomyces cerevisiae*.

Patient	Age/ Sex	ICU Stay	Underlying Disease and Medical Condition	Probiotic Use	Central Line	Previous Antibiotic Use	Other Positive Culture Specimen	Central Line Tip Culture	Co-Pathogen	Central Line Removal	Treatment	Outcome	Reference
1	F/66	NR	Metastatic gastric cancer Undergone chemotherapy	Yes	Yes	NR	None	NA	None	No	Fluconazole Voriconazole	Died	[27]
2	M/61	NR	Renal failure, hemodialysis, abdominal surgery	NR	Yes	NR	Central line	Positive	None	Yes	Miconazole 5-flucytosine	Died	[33]
3	F/71	NR	Aplastic anemia	NR	Yes	Yes	None	NR	Kluyveromyces marxianus	NR	Amphotericin B 5-flucytosine	Died	[34]
4	M/47	NR	Esophageal cancer	Yes	Yes	Yes	Central line	Positive	None	Yes	Fluconazole	Survived	[35]
5	M/50	Yes	Cardiac arrest	Yes	Yes	NR	None	NR	None	Yes	No	Died	[36]
6	F/51	Yes	Aortic surgery	Yes	Yes	NR	None	NR	None	Yes	Fluconazole	Died	[36]
7	M/60	Yes	ARDS	No	Yes	NR	Central line	Positive	None	Yes	Fluconazole	Survived	[36]
8	M/85	Yes	Respiratory failure	Yes	Yes	NR	None	NR	None	Yes	No	Survived	[36]
9	F/80	Yes	Respiratory failure	Yes	Yes	NR	None	NR	None	Yes	No	Survived	[36]
10	M/84	Yes	Peritonitis	Yes	Yes	NR	None	NR	None	Yes	Amphotericin B	Died	[36]
11	M/55	Yes	Stroke	Yes	Yes	NR	None	NR	None	Yes	No	Survived	[36]
12	M/34	Yes	Head trauma	Yes	Yes	Yes	None	Not performed	None	Yes	Fluconazole	Survived	[26]
13	M/48	Yes	Cerebral aneurysm	Yes	Yes	Yes	None	Not performed	None	Yes	Fluconazole	Survived	[26]
14	F/75	Yes	Myocardial infarction	Yes	Yes	Yes	Central line	Positive	None	Yes	Fluconazole	Survived	[26]
15	F/35	Yes	Multiple trauma	Yes	Yes	Yes	None	NR	None	Yes	NR	Survived	[26]
16	M/74	NR	Rheumatoid arthritis Heart surgery	Yes	Yes	Yes	None	Negative	None	Yes	Fluconazole	Died	[1]
17	F/48	NR	Chronic leukemia Allogenic stem cell transplantation	NR	Yes	NR	None	NR	None	Yes	Fluconazole	Survived	[37]

M, male; F, female; ICU, intensive care unit; NR, not reported.

## 4. Conclusions

We present a case of fungemia caused by *S. cerevisiae* in an immunocompromised patient with *C. difficile* colitis. Central line-related bloodstream infections caused by *S. cerevisiae* have rarely been reported. This case suggests that the use of probiotics should be approached with caution due to the risk of fungemia caused by *S. cerevisiae* in immunocompromised hosts with *C. difficile* colitis and central line placement. Early removal of the central line may be important for the elimination of fungemia in CRBSI due to *S. cerevisiae*, and clinical decisions regarding this should be balanced with the patient’s clinical needs.

## Data Availability

The original contributions presented in this study are included in the article. Further inquiries can be directed to the corresponding author.
